# A Case Report in Using a Laboratory-Based Decision Support Alert for Research Enrollment and Randomization

**DOI:** 10.1055/a-2702-1770

**Published:** 2025-10-24

**Authors:** April Barnado, Ryan P. Moore, Henry J. Domenico, Emily Grace, Sarah Green, Ashley Suh, Nikol Nikolova, Bryan Han, Allison B. McCoy

**Affiliations:** 1 Division of Rheumatology and Immunology, Department of Medicine, Vanderbilt University Medical Center, Nashville, Tennessee, United States; 2 Department of Biomedical Informatics, Vanderbilt University Medical Center, Nashville, Tennessee, United States; 3 Department of Biostatistics, Vanderbilt University Medical Center, Nashville, Tennessee, United States

**Keywords:** electronic health records and systems, clinical decision support, implementation and deployment, clinical care, precision medicine

## Abstract

**Objectives:**

Our objective was to identify barriers to implementing a custom clinical decision support (CDS) alert to randomize individuals in a pragmatic study, specifically those with a positive antinuclear antibody (ANA) test.

**Methods:**

We integrated a validated logistic regression model into the electronic health record to predict the risk of developing autoimmune disease for individuals with a positive ANA (titer ≥ 1:80). A custom CDS alert was created to randomize eligible individuals into a pragmatic study evaluating whether the risk model reduces time to autoimmune disease diagnosis. The custom CDS alert runs silently in the background and is not visible to providers. Individuals were randomized to either an intervention or control arm. In the intervention arm, the study team reviewed risk model results, notified providers of high-risk scores, and offered expedited rheumatology referrals to high-risk individuals in addition to standard of care. The control arm received standard care only. The study team accessed a daily Epic report containing randomization assignments and model variables.

**Results:**

Starting in June 2023, the risk model assessed 3,961 individuals and successfully randomized 2,105 individuals to date. Technical challenges that prevented the custom CDS alert from firing included an unanticipated change in the laboratory testing vendor and reporting due to a broken laboratory machine, followed by a change in the laboratory test name.

**Conclusion:**

This case report showcases the successful implementation of a laboratory-based custom CDS alert to randomize individuals for a pragmatic study. This approach enabled our study to be feasible across a large health care system. Key lessons learned included the importance of close collaboration with the laboratory team and thorough understanding of the laboratory testing, workflow, and reporting to ensure successful execution of the laboratory-based custom CDS alert.

## Background and Significance

Positive antinuclear antibodies (ANAs) cause diagnostic dilemmas for clinicians in all specialties. 1 2 3 While a positive ANA serves as a classification criterion for multiple autoimmune diseases, 14 to 27% of the general population has a positive ANA without having autoimmune diseases. 4 5 Currently, no tools exist to aide clinicians on deciding which individuals with positive ANAs to refer to rheumatology. Further, there is no standard triage system in rheumatology to handle positive ANA referrals. Positive ANAs are a frequent reason for rheumatology referral 1 6 in the setting of a national and international shortage of rheumatologists. 7 8 9 Typical wait times for rheumatology can be up to 6 to 12 months, based on anecdotal data from Southeastern U.S. academic medical centers. Long wait times can lead to delays in diagnosis and treatment that can result in irreversible disease damage. 1 10 11 12 13 14

We previously developed and validated a risk model to predict the risk of systemic autoimmune diseases in individuals with incident positive ANAs. 15 Model variables were selected based on clinical relevance, published systemic lupus erythematosus models, 16 17 and readily available data in the electronic health record (EHR). The logistic regression risk model includes age, sex, billing codes related to signs and symptoms for systemic autoimmune diseases (i.e., joint pain), and laboratory studies including ANA titer, platelet count, and having another autoantibody (i.e., dsDNA). The most important variables in the model included having another autoantibody, number of billing codes, and platelet count. The risk model has an area under the curve of 0.83 (95% confidence interval: 0.79–0.86).

Our institution has pioneered developing, validating, and deploying risk models in real-time within the EHR for venous thromboembolism, 18 19 20 postpartum hemorrhage, 21 readmissions, 22 and pressure injury. 23 The goal of the risk models is to identify individuals at high risk for an adverse outcome and then focus interventions on these individuals to improve patient outcomes. As resources are limited, high-risk individuals are prioritized for the intervention. To rigorously evaluate if the intervention affects the outcome, pragmatic studies are conducted where individuals are then randomized to either a control (standard care alone) or intervention arm (standard of care and incorporating risk model). Real-time randomization into these pragmatic studies is facilitated by a custom clinical decision support (CDS) alert.

### Project Overview

Once developed and validated, the risk model was deployed in the EHR in individuals with positive ANAs. To assess if the risk model could reduce time to autoimmune disease diagnosis, we created a custom CDS alert to randomize positive ANA individuals as part of an ongoing 2-year pragmatic study.

## Objectives

Our objective was to determine the barriers of implementing a silent custom CDS support alert to randomize individuals in a pragmatic study who had specific laboratory criteria, a positive ANA. The description of the development and validation of the ANA risk model and the performance of the model in the pragmatic study are beyond the scope of the current case report.

## Methods

**Fig. 1:**
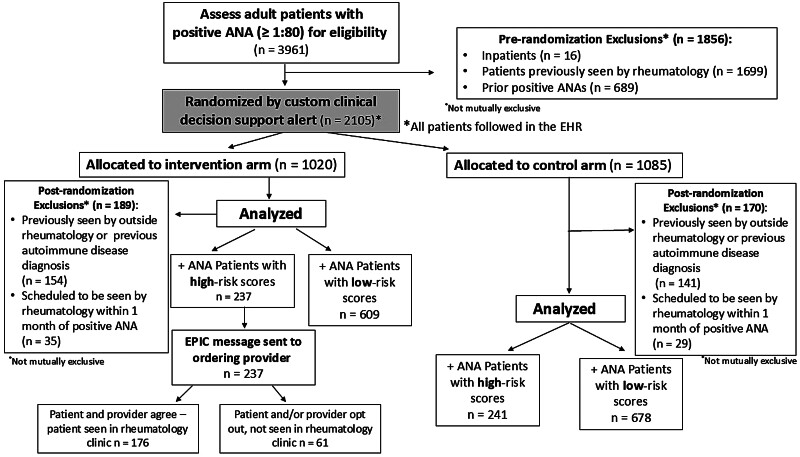
Consort diagram for patient enrollment in a randomized pragmatic study. All individuals with a positive antinuclear antibody (ANA), defined as a titer of ≥1:80 who did not meet prerandomization exclusions were randomized in a 1:1 fashion to either an intervention or control arm using a custom clinical decision support alert. For the intervention arm, risk model results were reviewed and acted upon by the study team in addition to standard of care. For the control arm, the risk score was calculated but not reviewed or acted upon with individuals receiving standard care only. Postrandomization exclusions are shown above. Only individuals randomized to the intervention arm with a high-risk score defined as a probability of ≥20% risk for developing a systemic autoimmune disease were targeted for an intervention. Specifically, ordering providers of the ANA test were sent an Epic message offering an expedited rheumatology evaluation.

We conducted a pragmatic study to evaluate whether a risk model could reduce time to autoimmune disease diagnosis among individuals with positive ANAs. A waiver of consent was approved due to minimal risk and study feasibility, as obtaining consent was not practical given the expected randomization of at least 2,000 individuals annually. Further, the study was deemed minimal risk as all individuals could receive standard of care (access to rheumatology referral). Following approval from the Institutional Review Board, we implemented a validated logistic regression model into the Epic EHR as a custom rule-based scoring system for individuals with positive ANAs (titer ≥ 1:80, via Hep-2 immunofluorescence assay). The ANA laboratory test result had the following two discrete components: (1) positive versus negative and (2) titer. A silent, background CDS alert automatically randomized eligible individuals with a titer of ≥ 1:80 to one of two study arms to evaluate whether the model reduces time to autoimmune disease diagnosis. Eligible individuals needed to meet the following criteria: ≥18 years old, not currently inpatient, no prior positive ANA in our health system, and not previously seen by our institution's rheumatology division ( Fig. 1 ). The CDS alert was configured to trigger only for individuals with a positive ANA result who met the above eligibility criteria. Upon triggering, the alert generated a random number to assign study arm, with assignment coded, saved, and made available only to the study team via Epic's Reporting Workbench. The Epic Reporting Workbench report was both updated and reviewed by a study team member daily. The custom CDS alert runs silently in the background and is not visible to providers.

Individuals were randomized by the custom CDS to either the intervention or control arm. For the intervention arm, risk model results were reviewed and acted upon by the study team in addition to standard of care. For the control arm, the risk score was calculated but not reviewed or acted upon with individuals receiving standard care only. Chart review was performed on all randomized individuals to identify postrandomization exclusions not easily captured in daily Epic reports. Postrandomization exclusions included individuals who had seen rheumatology outside of our institution, had prior diagnosis of autoimmune disease, or were scheduled to see rheumatology at our institution within 1 month of the positive ANA test. For individuals randomized to the intervention arm who had a high-risk score and no postrandomization exclusions, the study team sent secure Epic messages to the provider that ordered the ANA to notify them of a high-risk score. We then offered the provider and patient an expedited rheumatology clinic referral. A high-risk score was defined as a probability of ≥20% risk of developing systemic autoimmune disease in the next 10 years. This probability was chosen using Youden's cut-point, 24 a statistical method that maximizes sensitivity and specificity. For the primary outcome, we will compare time from positive ANA to autoimmune disease diagnosis in the intervention versus control arms, as part of an ongoing pragmatic study.

## Results

Starting in June 2023, the risk model was implemented in Epic with a custom CDS alert firing for individuals with positive ANA tests (titer ≥ 1:80). As of December 2, 2024, 3,961 individuals with positive ANAs were identified and 2,105 individuals were randomized. Of the 1,020 individuals randomized to the intervention arm, there were 237 individuals who had high-risk scores. These individuals had an Epic message sent to the ordering provider offering an expedited rheumatology evaluation, with 176 individuals evaluated in the rheumatology clinic.

### Technical Issues

Starting December 19, 2023, we observed that no new individuals with positive ANAs were being randomized in the daily Epic report. We learned that same day that part of the machine responsible for reporting the ANA titer had broken. Therefore, only the positive/negative component of the ANA test result was reported discretely in Epic with the ANA titer sent out to an external testing vendor. External testing for the ANA titer was reported in a PDF that was then scanned into the chart. As our custom CDS alert was dependent on the discrete ANA titer in Epic, individuals were not being randomized automatically. Over approximately 8 weeks, 305 individuals, of which 69 were high-risk and 39 were high-risk and randomized to the intervention arm, required manual randomization. Once the machine was fixed and ANA titers were again being performed at our institution and reported in Epic, automatic randomization in real-time resumed.

In April 2024, the central laboratory moved to an off-site location with equipment and processing changes. During this move, we observed that individuals were again not being randomized in our daily Epic report, with 82 individuals affected, including 26 high-risk and 11 high-risk individuals randomized to the intervention arm. The move not only introduced a new ANA testing system but also resulted in a change to the test's name and identifier. Thus, the existing coding failed to capture the updated laboratory test result. Once this new test was updated in the model build, individuals with positive ANAs resumed being randomized automatically.

## Discussion

We demonstrate the successful troubleshooting of a custom CDS alert to randomize individuals in a pragmatic study using the ANA test. The ANA test presents unique challenges, as it is initially reported as either positive or negative in a text format, followed by a titer that includes a combination of text, numeric characters, and symbols. The custom CDS alert enabled real-time study eligibility assessment and randomization for many individuals over the entire health system. Without the automatic assessment and randomization, the study may not have been feasible, as it would have required substantial labor for manual chart review. The pragmatic study of the ANA risk model is currently ongoing with model metrics and study outcomes to be presented in a future manuscript.

A recent study at our institution used a custom CDS alert to capture all pediatric inpatient admissions and to calculate a risk for developing a venous thromboembolism while hospitalized. 18 19 20 While the custom CDS alert for these studies and our study is not a traditional provider-facing alert, the custom CDS alert in our study enabled feasible and real-time enrollment and randomization for a pragmatic study across the health system. Outside of our institution, we identified studies that used a custom CDS alert to recruit individuals for studies 25 26 and used the EHR to randomize individuals through a dashboard 27 and a standard randomization tool. 28 29

When building a custom CDS alert to enroll and randomize individuals, particularly using a laboratory test, we advise careful consultation with the laboratory to understand the logistics and reporting of the laboratory test. In response to the technical issue in December 2023 that disrupted randomization, we worked closely with the director of the immunology laboratory to better understand the flow of ANA testing and reporting. This collaboration enabled a prompt response to the second technical issue reported above. Fortunately, we started manual randomization the first day of both technical issues discussed above. Thus, there were no delays in the clinical evaluation of high-risk individuals randomized to the intervention arm. Additional considerations include understanding how the laboratory test is presented (i.e., continuous or categorical variable) as well as the reference ranges and what values dictate a “positive” test for study inclusion. It is also important to understand the reporting of the laboratory test. For example, the laboratory testing may be conducted within the institution with results uploaded directly into the EHR. In contrast, the laboratory testing could be sent to an outside vendor with a paper copy being scanned as a PDF into the EHR. With this second scenario, it is imperative to understand how these results make their way back into the EHR and how the results are displayed. Lastly, it is helpful to consider that equipment and laboratory testing vendor could change during a study. These changes could alter the name of the laboratory test and affect the custom CDS alert firing that uses the “old” name of the test. Further, equipment and vendor changes could also potentially affect the “cutoff” value for what makes a laboratory test positive or negative, which could also alter the custom CDS alert firing.

Limitations of our study include silent custom CDS alert firing occurring only in our health care system, with portability to external sites not yet explored. We do not anticipate issues with use at other sites but do note the importance of communicating with a laboratory director who oversees ANA testing. Moving forward, we now meet quarterly with the immunology laboratory director to review any updates in laboratory testing. We recommend daily review of the Epic report, which was prespecified in our study protocol, as this allowed the study team to identify problems with randomization in real-time and to investigate promptly. While the silent custom CDS alert allows for automated randomization, currently chart review is performed for postrandomization exclusions, as these criteria could not easily be captured by queries. Future directions could include natural language processing methods to assess for relevant exclusions in clinic notes and reduce manual chart review.

## Conclusion

This case report demonstrates the successful implementation and troubleshooting of a laboratory-based custom CDS alert to randomize individuals for a pragmatic study. Laboratory-based custom CDS alert can help automate randomization to reduce manual chart review. Further, a laboratory-based custom CDS alert could also identify individuals in clinical scenarios in real-time across a large health system that need specific clinical monitoring or interventions. Understanding the laboratory testing, workflow, and reporting is key to ensuring successful execution of laboratory-based custom CDS alerts.

## Clinical Relevance Statement

Individuals with positive ANAs pose diagnostic dilemmas to clinicians across multiple specialties. No current guidelines exist on which individuals with a positive ANA should be referred to rheumatology to evaluate for autoimmune diseases. We developed and validated a risk model to function as a tool to predict risk of developing autoimmune diseases in individuals with positive ANAs. We then deployed and randomized our risk model in the EHR to assess if the risk model could reduce time to autoimmune diagnosis in individuals with positive ANAs. Our risk model is feasible as it uses a laboratory-based custom CDS alert to randomize individuals with positive ANAs.

## Multiple-Choice Questions

1. Which of the following is correct for interpreting positive antinuclear antibodies (ANAs)?
  - All patients with positive ANAs will develop autoimmune diseases.
  - All patients with positive ANAs need to be referred to rheumatology for evaluation.
  - All patients with positive ANAs need to be evaluated for symptoms for autoimmune diseases and then referred to rheumatology based on clinical suspicion for autoimmune disease.
  - None of the above.
  **Correct Answer** : The correct answer is option c. It is recommended to only order an ANA laboratory test in the setting of suspicion for autoimmune disease. While a positive ANA serves as a diagnostic criterion for multiple autoimmune diseases, the test alone only has an 11% positive predictive value for systemic autoimmune disease. Based on U.S. studies, approximately 17 to 24% of the general population has a positive ANA without having autoimmune disease.
2. When using a laboratory test as a criterion to enroll patients in a study, which of the following is important to understand about the laboratory test?
  - Variable type of the laboratory test (categorical vs. continuous).
  - Reference range (cutoff values).
  - Location and display of the laboratory test in the her.
  - All of the above.
  **Correct Answer** : The correct answer is option d. All of the above are important considerations when using a laboratory test to enroll subjects into a study. It is important to understand how the variable is expressed (categorical variable with text or symbols vs. a continuous variable). It is also important to understand its reference range and what dictates a positive or a negative test. Lastly, it is important to understand how and where the laboratory test is displayed in the EHR.
3. When troubleshooting a laboratory-based best practice alert, which of the following is important to consider?
  - Laboratory test reference range.
  - Laboratory test workflow.
  - Laboratory test reporting.
  - All of the above.
  **Correct Answer** : The correct answer is option d. All of the above are important considerations for troubleshooting why a laboratory-based BPA may or may not be firing properly. Laboratory testing can change during the course of the study with a new vendor or process potentially affecting the laboratory test reference range, workflow, and/or result reporting.
